# Application of electrochemical biosensors in tumor cell detection

**DOI:** 10.1111/1759-7714.13353

**Published:** 2020-02-26

**Authors:** Zhenhua Zhang, Qingchao Li, Xin Du, Min Liu

**Affiliations:** ^1^ Institute of Biomedical Sciences, Shandong Provincial Key Laboratory of Animal Resistance Biology, Collaborative Innovation Center of Cell Biology in Universities of Shandong, College of Life Sciences Shandong Normal University Jinan China

**Keywords:** Biosensor, detection, electrochemical, tumor cell

## Abstract

Conventional methods for detecting tumors, such as immunological methods and histopathological diagnostic techniques, often request high analytical costs, complex operation, long turnaround time, experienced personnel and high false‐positive rates. In addition, these assays are difficult to obtain an early diagnosis and prognosis quickly for malignant tumors. Compared with traditional technology, electrochemical technology has realized the study of interface charge transfer behavior at the atomic and molecular levels, which has become an important analytical and detection tool in contemporary analytical science. Electrochemical technique has the advantages of rapid detection, high sensitivity (single cell) and specificity in the detection of tumor cells, which has not only been successful in differentiating tumor cells from normal cells, but has also achieved targeted detection of localized tumor cells and circulating tumor cells. Electrochemical biosensors provide powerful tools for early diagnosis, staging and prognosis of tumors in clinical medicine. Therefore, this review mainly discusses the development and application of electrochemical biosensors in tumor cell detection in recent years.

## Introduction

Tumors, as a nonhereditary genetic disease, can be divided into benign and malignant tumors, the latter can metastasize, grow rapidly, and produce harmful substances, thereby seriously threatening human health. In addition, malignant tumors (also named cancers) have developed a variety of genetic mechanisms to adapt to the stresses of living environment through genetic mutations, thereby escaping growth inhibition signals and immune surveillance systems.^1^, ^2^ During the evolution from normal cells to tumor cells, there are specific proteins or small molecules used as markers for tumor diagnosis on the cell surface or in the serum, which brings good gospel for the early diagnosis and treatment of tumors.^3^ For a long time, histopathological diagnosis has been the gold standard for cancer diagnosis and the basis for clinical treatment.^4^ However, histopathological diagnostic techniques have the disadvantages of high analytical costs, complex operations, long turnaround time, and high false‐positive rates, and it is difficult for them to meet the requirements for early diagnosis and prognosis of malignant tumors. Fluorescence imaging combined with confocal microscopy can directly observe the rich location information of cancer cells.^5^, ^6^, ^7^ However, the technology cannot meet the requirements of high sensitivity measurement. Therefore, the development of new tools is in demand. Recent studies have highlighted an electrochemical technique which has been proven to have ultra‐high sensitivity and accuracy in the quantitative detection of breast, prostate, liver and cervical cancer cells.^8^, ^9^, ^10^

The most classical application of electrochemical biosensors in the early diagnosis of tumors is the detection of tumor cells by biosensors based on cell impedance sensing technology. Cyclic voltammetry (CV), as a commonly used electrochemical research method, can be used to judge the microscopic reaction process on the electrode surface, so as to detect the change in impedance or microcurrent at the electrode interface caused by the growth of cells on the electrode surface. Differential pulse voltammetry (DPV) is a method based on linear sweep voltammetry and staircase voltammetry which has a lower background current and higher detection sensitivity. In addition, it displays the highly stable and specific capture of cancer cells by producing nontoxic biological modifications on the working electrodes of electrochemical biosensors, such as with covalently linked biotin, monoclonal antibodies, lactoglobulin A and aptamer. Therefore, the detection of tumor cells without lysis and fixation is made possible, which simplifies the analysis process and improves the accuracy of the results. Here, we review the latest developments in electrochemical biosensors for the detection of tumors (Table 1). We highlight four aspects: electrochemical biosensor in tumor cell detection; electrochemical immunosensors in tumor cell detection; electrochemical nucleic acid biosensors in tumor cell detection and detection of circulating tumor cells (CTCs).

**Table 1:** Detection of tumor cells using electrochemical biosensors

Analyte	Detection technique	Nanomaterials	Performance	Reference
MCF‐7	Electrochemical impedance	Au nanoparticles (AuNPs)	LOD: 10 cells/mL	Wang *et al*.^11^
Hela	Electrochemical impedance	Multiwall carbon nanotubes (MWCNTs)	Linear range: 2.1 x 10^2^–2.1 x 10^7^ cells/mL LOD: 70 cells/mL	Liu *et al*.^12^
HL‐60	Cyclic voltammetry (CV) Electrochemical impedance Differential pulse voltammetry (DPV)	Multiwall carbon nanotubes (MWCNTs)	Linear range: 2.7 x 10^2^–2.7 x 10^7^ cells/mL LOD: 90 cells/mL	Xu *et al*.^13^
K562	Cyclic voltammetry (CV) Electrochemical immunosensors	Au nanoparticles (AuNPs)	Linear range: 1.0 x 10^2^–1.0 x 10^7^ cells/mL	Ding *et al*.^14^
MCF‐7	Electrochemical nucleic acid biosensors	DNA‐AgNC	LOD: 3 cells/mL	Cao *et al*.^15^
MCF‐7	Electrochemical nucleic acid biosensors	Multiwall carbon nanotubes (MWCNTs)	Linear range: 1.0 x 10^2^–1.0 x 10^7^ cells/mL LOD: 25 cells/mL	Yazdanparast *et al*.^16^
CTCs	Cyclic voltammetry (CV) Electrochemical impedance	Pt@Ag nanoflowers AuNPs/Acetylene black	Linear range: 20–10^6^ cells/mL LOD: 3 cells/mL	Tang *et al*.^17^
CTCs	Cyclic voltammetry (CV) Differential pulse voltammetry (DPV) Electrochemical impedance	Magnetic Fe3O4 nanospheres (MNs) Cu2O nanoparticles (Cu2O NPs)	Linear range: 3.0–3000 cells/mL LOD: 1 cells/mL	Luo *et al*.^18^
CTCs (MCF‐7)	Cyclic voltammetry (CV) Electrochemical impedance	Ni micropillars/ PLGA electrospun nanofbers	Linear range: 10–10^5^ cells/mL LOD: 8 cells/mL	Wu *et al*.^19^
K562	Differential pulse voltammetry (DPV)	Graphene oxide/ quantum dots (QDs)	LOD: 60 cells/mL	Zheng *et al*.^20^
CTCs	Cyclic voltammetry (CV) Electrochemical impedance		Linear range: 1.0 x 10^2^–1.0 x 10^5^ cells/mL LOD: 25 cells/mL	Wang *et al*.^21^
HepG2	Electrochemical impedance	Carbon nanotubes (CNTs)	Linear range: 10–10^5^ cells/mL	Liu *et al*.^22^
CTCs	Cyclic voltammetry (CV) Electrochemical impedance		Linear range: 30–10^6^ cells/mL LOD: 10 cells/mL	Shen *et al*.^23^
HT 29 FR‐positive cancer cells	Electrochemical	Functionalized fibrous Nanosilica (KCC‐1)	Linear range: 50–1 x 1.2 x 10^4^ cells/mL LOD: 50 cells/mL	Soleymani *et al*.^24^

## Electrochemical biosensor in tumor cell detection

**Figure 1:**
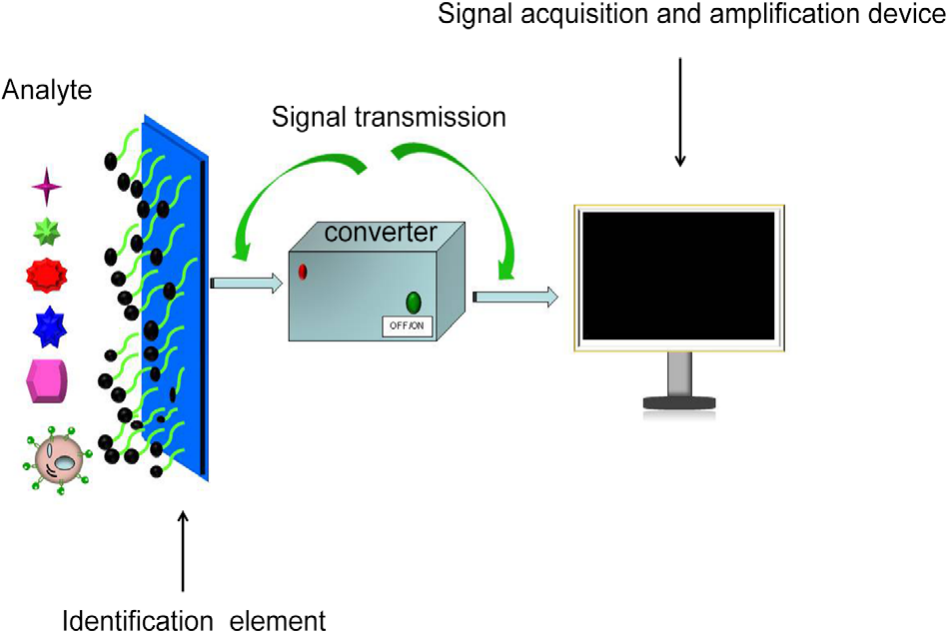
The recognition system selectively interacts with the analyte and converts the resulting chemical parameters into a certain signal. The transduction system receives the signal and transmits it to the electronic system in the form of an electrochemical signal, and the electronic system further amplifies the output.

**Figure 2:**
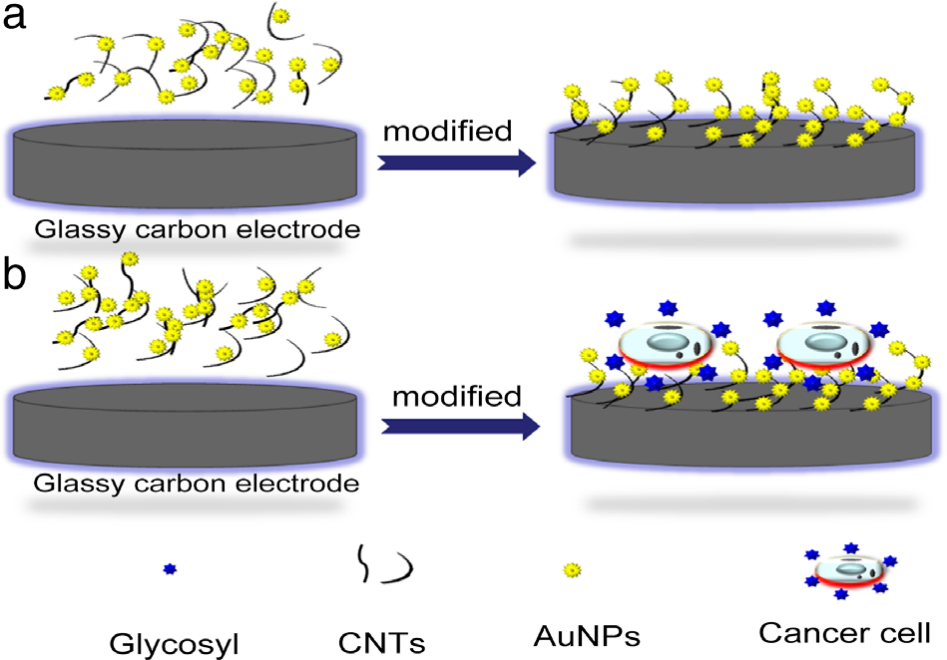
Cells that grow on the surface of microelectrodes can change the impedance at the interface of the electrode, thus obtaining biological information related to the physiological functions of the cells. (**a**) Glassy carbon electrodes are modified by composite materials composed of carbon nanotubes (CNTs) and gold nanoparticles (AuNPs) to improve their sensitivity and detection range (control). (**b**) Cancer cell is adhered to a composite modified glassy carbon electrode, and the change in cell layer resistance is detected (experiment).

The electrochemical biosensor consists of an identification system and a transduction system. The function of the identification system is to selectively interact with the analyte and convert the resulting parameters into a certain signal. The function of the transduction system is to receive signals and transmit them to the electronic system in the form of electrochemical signals. The electronic system further amplifies and outputs, realizing the quantification and research of the analyte (Fig 1). Because of the advantages of good selectivity, high sensitivity, simple equipment and low price, the electrochemical biosensor has been widely used in many fields, including food testing, environmental monitoring, clinical medicine, animal disease detection and drug screening.^25^, ^26^, ^27^, ^28^, ^29^, ^30^ One of the most classical techniques in electrochemical biosensor technology is cell impedance sensing. It is based on the principle that cells growing on the surface of a microelectrode can change the impedance at the interface of the adherent electrodes, where biological information related to the physiological functions of cells can be obtained. Therefore, an electrochemical biosensor based on cell impedance sensing technology can measure changes in cell layer resistance caused by cell morphology, cell movement, or cell contact, which can monitor cell dynamic behavior in real time quantitatively without damage (Fig 2). Based on the above principles, researchers have constructed various electrochemical impedance sensors with modified working electrodes based on the innovative technique of capturing tumor cells, and the detection of the following human tumor cells has been demonstrated: HeLa, MCF‐7 (breast cancer), HL‐60 (human promyeloacute leukemia cell line), HCT‐116 (human colorectal cancer cells) and HepG2 (hepatocellular carcinomas).^11^, ^12^, ^13^, ^22^, ^31^, ^32^, ^33^

The most important step in building the cell electrochemical sensor is to fix the cells onto the surface of the working electrode to produce electrochemical signals. The traditional fixing method has some disadvantages, such as low survival rate, poor stability, additional increase in diffusion resistance and so on.^34^, ^35^ As a kind of biosensor‐fixed matrix with good biocompatibility, no toxicity, superior protein adsorption and gel‐forming ability, gelatin is widely used in the construction of living cell‐fixed and bionic interface. For example, Zhu *et al*. obtained c‐SWCNTs‐AuNPs‐gelatin nanoparticles by ultrasonic assembly using gelatin, gold nanoparticles and single‐walled carbon nanotubes and successfully achieved in vitro fixation and highly sensitive electrochemical detection of human leukemia HL‐60 cells.^36^ Li and coworkers wrapped gelatin on the surface of Au nanoflowers (AuNFs) to produce AuNFs@gelatin and combined it with cationic conjugated materials to fix and image HeLa cells.^37^

Sensitive biosensors based on various electrochemical techniques have been widely used in the detection of cancer cells. In 2017, Feng *et al*. gave a detailed review of electrochemical detection of tumor cells, and summarized the analytical performance of biosensors used to detect cancer cells. In recent years it has been pointed out that the integration of electrochemical sensors into mobile sensitive detection devices is a widely explored direction in the future.^38^ In addition, we still need to develop new electrochemical probes using new nanobiomaterials to improve the capture rate, develop new nanomaterial‐modified electrodes to improve conductivity, and detect cancer cells with high sensitivity in a short time in a simple way.

## Electrochemical immunosensors in tumor cell detection

**Figure 3:**
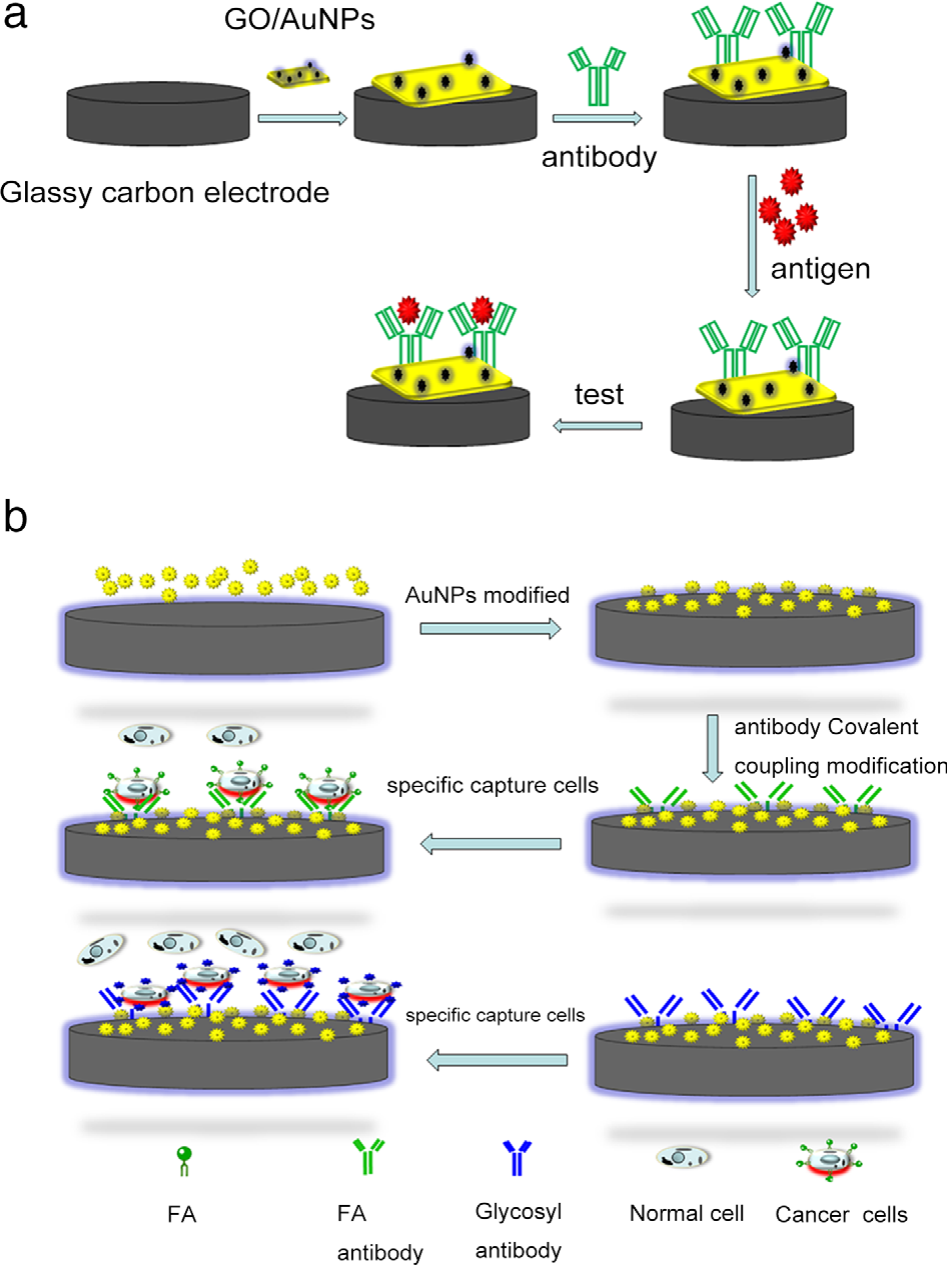
Schematic diagram: (**a**) Electrochemical immunosensor. (**b**) Specific antibody is covalently linked to a modified glassy carbon electrode for specific capture of cancer cells.

An electrochemical immunosensor is a product based on the combination of antigen‐antibody specific reaction and electrochemical technology. The basic principle is that the antigen‐antibody, as a molecular recognition element, is in direct contact with the electrochemical sensing element and converts the signal of a certain or a certain kind of chemical concentration into the corresponding electrical signal through the sensing element (Fig 3a). Therefore, biosensors constructed on the basis of specific reactions between antigens and antibodies, specific recognition function of adapters and the cell impedance principle have successfully achieved high‐sensitivity linear detection of various cancer cells, including human liver cancer cell line HepG2, human breast cancer cell line MCF‐7, small cell lung cancer, lung adenocarcinoma, squamous cell cancer, skin cancer, prostate cancer and breast cancer.^39^, ^40^, ^41^ In addition, glycosyls on the cell surface play an important role in cell recognition, adhesion, immune response, and the occurrence and migration of cancer cells.^42^, ^43^, ^44^ Therefore, the cell electrochemical sensor based on surface‐specific glycosyls of cancer cells has also successfully achieved the specific recognition and linear detection of HeLa cells, polysaccharide‐rich K562 leukemia cells, and MDA‐MB‐231 cells.^14^, ^45^, ^46^, ^47^, ^48^

Folic acid (FA) receptor is a cell surface receptor that is excessively expressed on most human tumor cells and rarely expressed or not at all in normal organs. Therefore, FA is often used as a target for antitumor drugs.^49^, ^50^, ^51^, ^52^ Using the high affinity of FA and FA receptors, researchers used hydrothermal synthesis of functionalized fiber nanosilica (KCC‐1), which was then functionalized with FA molecules to produce KCC‐1‐NH2‐FA nanoparticles. Based on the excellent bleaching stability and excellent surface area to volume ratio of KCC‐1‐NH2‐FA nanoparticles, a more sensitive cell sensor was designed for the detection of cancer cells HT‐29 with a detection range of 50 to 1.2 x 10^4^ cells/mL and the lower limit of detection is 50 cells/mL (Fig 3b).^24^

As shown in Table 2, we briefly summarize the common and hazardous tumor markers, apart from the tumor markers mentioned in the article, the remaining tumor markers and tumor markers to be discovered provide broad prospects for the specific detection of tumor cells by electrochemical immunosensors. However, in the preparation of electrochemical immunosensors, the fixation of antigen and antibody is an important factor affecting the performance of the sensor, which directly affects the service life, reproducibility and detection limit of the sensor. Due to the specific reaction of antigens and antibodies, electrochemical immunosensors have higher specificity and selectivity than other biosensors, and have been widely used and applied.

**Table 2:** Summary of the common and serous tumor markers

Tumor cells	Cell‐surface/serum markers	Reference
Liver cancer stem cell	CD13	Sun *et al*.^53^
Hepatocellular carcinoma	Assessing serum α‐fetoprotein (AFP) Des‐γ‐carboxyprothrombin (DCP) AFP‐L3 Glypican‐3 (GPC3) Golgi protein‐73 (GP73)	Tsuchiya *et al*.^54^
Lung cancer cell line	Carbonic anhydrase 9 (CA9) G protein‐coupled receptor 87 (GPR87) LYPD3 SLC7A11 CXorf61	Cohen *et al*.^55^
Breast cancer cell line	Human epidermal growth factor receptor 3 (HER‐3)	Lv *et al*.,^56^ Chiu *et al*.^57^
Breast cancer cell line	Carbohydrate antigen125 (CA125) Human epidermal growth factor receptor‐2 (Her‐2) Cytokeratin5/6 (CK5/6) E‐cadherin (E‐cad) carcinoembryonic antigen (CEA) MUC1	Liu *et al*.,^58^ Jafari *et al*.^59^
Gastric cancer cell line	Folic acid (FA) GRP78 anti‐CD146 MAb BRCAA1 MAb	Liu *et al*.^60^
Acute myeloid leukemia (AML)	CD123, CD45, CD34, CD38, MLL‐AML, core binding factor, among others	Prada‐Arismendy *et al*.^61^
HT‐29	Folic acid (FA)	Soleymani *et al*.^24^

## Electrochemical nucleic acid biosensors in tumor cell detection

**Figure 4:**
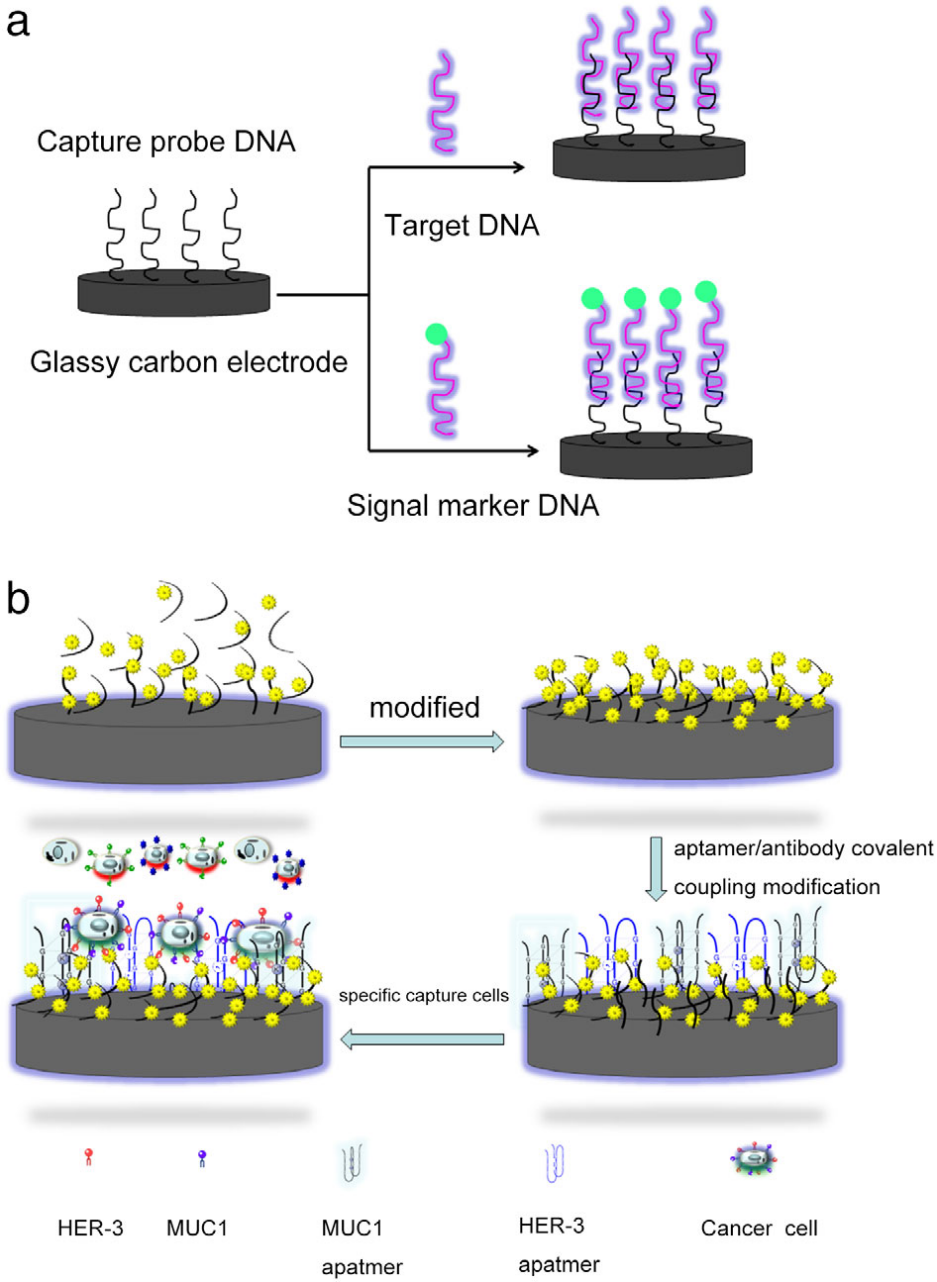
Schematic diagram: (**a**) Electrochemical nucleic acid biosensors. (**b**) Specific aptamers is covalently linked to the modified glassy carbon electrode to enhance the specificity of cancer cell capture.

Electrochemical nucleic acid biosensors use nucleic acid molecules as molecular recognition elements, whose principle is to fix a single strand of oligonucleotides on the electrode and hybridize with the target DNA, and detection of target substances by detecting changes in electrochemical parameters before and after hybridization. The target substances can be DNA, miRNA, or other biological molecules (Fig 4a). Aptamer is a synthetic nucleic acid with high specificity and affinity, and ease of biological and chemical modification that has been screened by the screening technique SELEX in vitro (Systematic Evolution of Ligands by Exponential Enrichment). A nucleic acid adapter shows highly specific binding to tumor cell surface target molecules and is easy to use, which has been widely used in the construction of cell sensors in recent years,^62^, ^63^, ^64^, ^65^ greatly improving the target recognition ability of sensors and detection selectivity to tumor cells.^66^, ^67^, ^68^, ^69^ For example, Li *et al*. used MUC1 to bind an aptamer for detecting MUC1 proteins on the surface of tumor cells while identifying their CEA proteins with nanometer CdS‐labeled carcinoembryonic antigen (CEA), which effectively reduced the occurrence of false positives in the detection of tumor cells.^58^ Studies have shown that ITO electrodes with good light transmittance and electrical conductivity were first modified by the AS1141 aptamer, which can selectively bind to the overexpressed nucleolins on the surface of breast cancer cells McF‐7. Then, the mucin‐1 antibody (anti‐muc1) and DNA‐AgNCs complexes with unique fluorescent and electrochemical properties template silver nanocluster (DNA‐AgNCs) bind to the MUC1 on the surface of McF‐7 cells to form the sandwich structure. The strong red fluorescence of DNA‐AgNCs shows the presence of cancer cells, the strong conductivity of DNA‐AgNCs can improve the sensitivity of quantitative analysis, making the detection limit up to 3 cells/mL.^15^ In addition, the sensitivity and specificity of tumor cell detection can be improved by using the electrochemical sensor constructed by a dual‐aptamer. For example, human epidermal growth factor receptor 3 (HER‐3) binding aptamer and MUC1 aptamer were used to simultaneously detect MUC1 protein and HER‐3 receptor on the surface of breast cancer cells, which not only kept the probe stable in the complex system, but also had good selectivity and sensitivity for the detection of MCF‐7 cells. The linear calibration range of this electrochemical method was 1.0 x 10^2^ to 1.0 x 10^6^ cells/mL, and the detection limit 100 cells/mL (Fig 4b).^56^ The research group of Qu *et al*. directly combined the aptamer TLS1c through the flexible linker and the aptamer TLS11a through the rigid linker to the surface of glassy carbon electrode (GCE) to capture MEAR cancer cells; the linear range of detection was 1–14 MEAR cells, and the detection limit was 1 MEAR cell in 10 whole blood cells.^70^ In the study by Yazdanparast *et al*. biocompatible nanocomposite consisting of multiwall carbon nanotubes (MWCNT) and poly(glutamic acid) was placed on a glassy carbon electrode (GCE), then a mucin 1 (MUC1)‐binding aptamer was first immobilized on the surface of modified GCE. In order to enhance the selectivity, another aptamer (labeled with silver nanoparticles) was used for secondary MCF‐7 cell recognition, which building a kind of electrochemical sensors with high selectivity, sensitivity, stability and reproducibility. Under optimal conditions, the detection range of the sensor was 1.0 x 10^2^ to 1.0 x 10^7^ cells/mL, and the detection limit was 25 cells/mL.^16^

**Figure 5:**
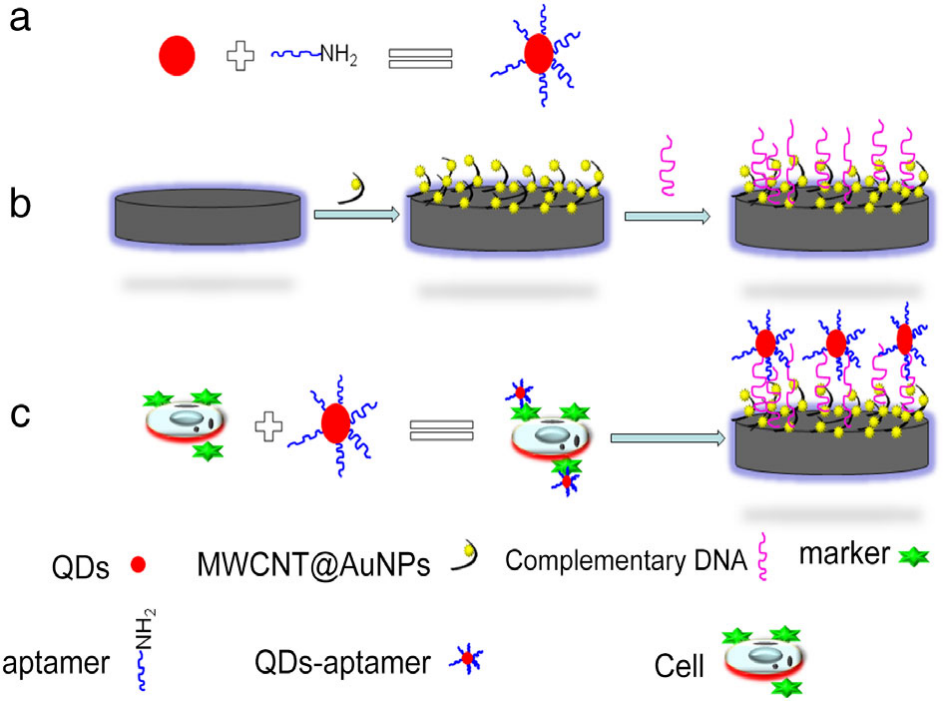
Electrochemical probes based on quantum dot‐aptamers were constructed to detect tumor cells. (**a**) Construction of electrochemical probe. (**b**) Working electrode was modified by nanomaterials. (c) The probe captures the tumor cells. Nitric acid digests probes that specifically bind to tumor cells for electrochemical detection.

Quantum dots (QDs), also known as semiconductor nanocrystal, has been a hot new type of luminescent nanomaterials in recent years, with unique optical and electrical properties. In addition, QDs has active electrochemical properties, and its metal components can show very sharp redox peak signals after voltammetry analysis. Therefore, QDs can be used as an electroactive substance with signal amplification for the construction of a variety of biosensors. In recent years, for electrochemical cell sensors, QDs and aptamer are usually combined to capture cells, and electrochemical analysis of the metal components of QDs is used to achieve the purpose of quantitative detection of cells (Fig 5). For example, in 2011, Zhu *et al*. directly assembled complementary DNA (cDNA), aptamer and QDs onto the surface of the gold electrode to achieve highly sensitive detection of tumor cells with a detection limit of 100 cells/mL^.^ 71 In recent years, in order to achieve high selectivity and sensitivity of cancer cells to capture and detect, glassy carbon electrodes were firstly modified by multiwall carbon nanotubes (MWCNT), gold nanoparticles (AuNPs), polydopamine (PDA), graphene oxide (GO), polyaniline (PANI) and concanavalin A (Con A) using a layer‐by‐layer technique. Subsequently, the aptamer‐DNA concatamer‐CdTe quantum dots (QDs) as the signal amplification probe was covalently connected to the surface of the modified electrode, making a sensor with high stability, selectivity and sensitivity, with a detection limit reaching 50–60 cells/mL.^20^, ^72^

The detection technology of tumor cells based on an aptamer electrochemical probe has been widely used. In 2019, Chen *et al*. published a detailed review of the application of aptamer‐based electrochemical cytosensors in tumor diagnosis.^73^ However, in vitro screening technique SELEX is still needed to obtain more specific aptamers to improve the detection range and limit of electrochemical sensors.

## Detection of circulating tumor cells by electrochemical biosensors

Circulating tumor cells (CTCs) were first discovered in 1869 by Ashworth who found similar tumor cells in peripheral blood during an autopsy of a patient who died of cancer. The main cause of death from malignant tumors is when tumor cells are released from a primary or metastatic lesion into the peripheral blood or lymphatic circulation, resulting in CTCs and metastasis in other parts. Previous studies have shown that CTCs can be a new diagnostic target for tumor staging and prognosis (fewer CTCs indicate longer survival) and can provide information for treatment evaluation.^74^, ^75^, ^76^, ^77^ Therefore, it is becoming more and more important to detect CTCs rapidly and accurately in peripheral blood for the clinical treatment of tumors and prognosis.

As a fast and efficient detection tool, electrochemical biosensors have been widely used in the detection of tumor cells. However, the average density of tumor cells in the blood is 200 cells/mL, which accounts for only 0.004% of the number of cells. Therefore, it is necessary to develop highly specific and sensitive tools to capture CTCs at low concentrations in the blood. In 2014, Costa *et al*. reviewed isolation and detection with high sensitivity to circulating tumor cells (CTCs) through various biosensors, and pointed out that compared with other biosensors, an electrochemical biosensor has higher sensitivity, simplicity and low cost. Different new nanomaterials are being used to modify electrodes to amplify biometric event signals, narrow detection range and improve detection sensitivity; at the same time, more specific electrochemical probes need to be constructed to improve the ability to capture circulating tumor cells (CTCs); both are still challenges for researchers.^78^ For example, in the report by Shen *et al*. a label‐free electrochemical impedance biosensor based on the specific recognition between specific epithelial cell adhesion molecules (EpCAM) overexpressed on the cell membrane and EpCAM aptamer was constructed to detect CTCs. First, 6‐mercapto 1‐hexanol (MCH) was fixed on the gold electrode; second, the capture probe was directionally inserted in MCH interspaces; the detection range of the sensor was 30 to 10^6^ cells/mL, and the detection limit was 10 cells/mL.^23^ Tang *et al*. designed a novel ultrasensitive immunoassay protocol by using Pt@Ag nanoflowers (pt@AgNFs) and AuNPs/Acetylene black (AuNPs/AB) nanomaterial to detect CTCs.^17^ Pt@AgNFs had high specific surface area and good biocompatibility, and were not only used as the carriers of signal antibodies (Ab_2_) but also catalyzed the reduction of H_2_O_2_ to amplify the current signal. AuNPs/AB nanomaterial was used as a substrate material to increase the specific surface area and conductivity of the gold electrode. The detection range of the sensor was 20 to 1.0 x 10^6^ cells/mL, and the detection limit was 3 cells/mL.^17^ In addition, researchers have cleverly combined photoexcitation and electrochemical detection processes to construct photoelectrochemical (PEC) biosensors with higher detection sensitivity for the detection of circulating tumor cells. In this study, a PEC biosensor was proposed based on hexagonal carbon nitride tubes (HCNT) as photosensitive material, and magnetic Fe3O4 nanospheres were used for efficient magnetic capture of CTCs, and Cu2O nanoparticles were used for signal amplification, making the detection range of this sensor range from 3 to 3000 cells/mL, with a detection limit down to 1 cell/mL.^18^

With the development of science and technology, the basic operating units such as sample preparation, reaction, separation and detection of biological, chemical, and medical analysis processes are integrated into microscale chips, which automatically completes the entire analysis process and combines with electrochemical sensing for the detection of CTCs. In 2007, Nagrath and coworkers used a microfluidic chip modified with an epithelial‐specific adhesion molecule (EpCAM) antibody to successfully separate untreated peripheral blood CTCs for the first time.^79^ At present, the microfluidic chip based on an antibody as the trapping probe has successfully detected CTCs.^80^, ^81^, ^82^, ^83^ At the same time, as an artificial small molecule that is easier to preserve and modify than antibodies, the aptamer is more suitable for functional modification of microfluidic chips. For example, the microfluidic chip modified by nucleic acid aptamer sgc8 has successfully achieved the separation and capture of target cells from many samples with a capture efficiency of 80% and a specificity greater than 97%. In addition, different aptamers can be used to separate and capture different target cells.^84^, ^85^ On the basis of earlier studies, Soper *et al*. and Tsing *et al*. used aptamers that identified PSMA and A549 cells to perform functional modifications on microfluidic chips, and constructed microfluidic devices to capture tumor cells, and they were able to detect CTCs in the blood of cancer patients.^86^, ^87^ However, in these studies, the length of the aptamers modified on the surface of the microfluidic chip exposed to the solution was only a few nanometers, which makes microfluidic chip detection inefficient and difficult to capture cells in high‐speed flowing liquid. To overcome this shortcoming, researchers continue to explore the use of cyclic DNA templates and connected primers at the end of the template, and then in the presence of polymerase and dNTP, each primer extends along the cyclic DNA template to finally generate a single‐stranded DNA consisting of a plurality of aptamers in series as a capture probe, which can effectively enhance its ability to capture the CTCs.^21^

With the rapid development of micro/nano manufacturing technology, the analysis method based on three‐dimensional (3D) bionic interface has become a hot research topic in nanotechnology and life sciences. Micro/nanostructure‐based devices have been identified as the simplest and most effective technologies for capturing CTCs. Chen and coworkers showed a nickel (Ni) microcolumn cell sensor deposited by electro‐textile nanofibers. First, ultralong poly (lactic‐co‐glycolic acid) (PLGA) nanofibers were laterally stacked on the surface of nickel micropillars by electrospinning to construct a 3D biomimetic interface for capturing CTCs, which would be connected with quantum dot (QD). The functionalized anti‐EpCAM antibody (QD‐EpCAM) was modified at the 3D biomimetic interface to successfully achieve the highly specific detection of MCF7 breast cancer cells as a CTC model. The detection range was 10^1^ to 10^5^ cells/mL, and the detection limit was 8 cells/mL.^19^ In summary, the combination of electrochemical sensing technology and microfluidic chip technology can provide a powerful, rapid and easy‐to‐use tool for the clinical detection of CTCs.

## Conclusions and perspectives

Compared with normal cells, tumor cells, especially malignant tumors, exhibit abnormal movement and migration capabilities, rapid cell division, and cytoskeletal abnormalities.^88^, ^89^, ^90^ At the same time, existing studies have shown that persistent inflammation can also trigger and exacerbate malignant tumors.^91^ These characteristics not only give researchers new ideas on how to treat cancer; for example, by inhibiting the activity of microtubule motor proteins, blocking mitosis or development of inflammatory factor‐related inhibitors (such as histone deacetylase 6 [HDAC] inhibitors), achieving the purpose of anticancer agents, they also provide valuable reference to assist with the early diagnosis of tumors.^63^, ^92^, ^93^ Electrochemical biosensor technology, as a new type technology of tumor detection, has achieved breakthrough results after decades of development. In particular, the emergence of cell electrochemical sensors provides convenient tools for cell counting, cell classification, and the detection of tumor cells.^94^, ^95^ Among the many breakthrough results, the detection of tumor cells has not only achieved high sensitivity (limit of detection of 10 tumor cells in 250 μL samples^96^) and high specificity, but has also made it possible to detect double antigen on the tumor cell surface, successfully avoiding false positive results. However, in the process of sensor configuration and application research, some deficiencies and improvements have also been found.

First, with the advent of nucleic acid adapters and microfluidic chips, the problem of efficient and specific trapping of tumor cells has been solved. However, during the application of the electrochemical sensors, an irreversible chemical reaction occurs between recognition elements and target on the surface of tumor cells, or the recognition element is contaminated with blood impurities which greatly reduces the reuse rate of the sensors and recognition ability of the identification elements. Therefore, the recognition elements of the sensors are chemically treated with different regenerative solvents in different situations to restore the recognition function in time for the purpose of reuse.

Second, in the preparation of nanomaterials and the preparation of functionalized nanocomposites, nanomaterials with high catalytic properties should be combined with carbon nanotubes and peptide carbon nanotubes to improve sensitivity, selectivity and stability of nanocomposites. In terms of biocatalytic induction of nanomaterials, a new biosensor interface should be explored further for the assembly of biomolecules and nanometer microarray so as to achieve high specificity and high sensitivity detection of CTCs in complex blood samples. In addition, the manufacturing process of future sensors could be more delicate, which will not only improve the performance of future sensors but will also promote the miniaturization of sensors to meet the needs of specific situations.

## Disclosure

The authors have no conflicts of interest to declare.
